# Deep sequencing of the Mexican avocado transcriptome, an ancient angiosperm with a high content of fatty acids

**DOI:** 10.1186/s12864-015-1775-y

**Published:** 2015-08-13

**Authors:** Enrique Ibarra-Laclette, Alfonso Méndez-Bravo, Claudia Anahí Pérez-Torres, Victor A. Albert, Keithanne Mockaitis, Aruna Kilaru, Rodolfo López-Gómez, Jacob Israel Cervantes-Luevano, Luis Herrera-Estrella

**Affiliations:** Laboratorio Nacional de Genómica para la Biodiversidad-Langebio/Unidad de Genómica Avanzada UGA, Centro de Investigación y Estudios Avanzados del IPN, 36500 Irapuato, Guanajuato Mexico; Red de Estudios Moleculares Avanzados, Instituto de Ecología A.C., 91070 Xalapa, Veracruz, Mexico; Investigador Cátedra CONACyT en el Instituto de Ecología A.C., Veracruz, Mexico; Department of Biological Sciences, University at Buffalo, Buffalo, NY 14260 USA; Department of Biology and Center for Genomics and Bioinformatics, Indiana University, Bloomington, IN 47405 USA; Department of Biological Sciences, East Tennessee State University, Johnson City, TN 37614 USA; Department of Biomedical Sciences, East Tennessee State University, Johnson City, TN 37614 USA; Instituto de Investigaciones Químico-Biológicas (IIQB), Universidad Michoacana de San Nicolás de Hidalgo, 58030 Morelia, Michoacán Mexico

## Abstract

**Background:**

Avocado (*Persea americana*) is an economically important tropical fruit considered to be a good source of fatty acids. Despite its importance, the molecular and cellular characterization of biochemical and developmental processes in avocado is limited due to the lack of transcriptome and genomic information.

**Results:**

The transcriptomes of seeds, roots, stems, leaves, aerial buds and flowers were determined using different sequencing platforms. Additionally, the transcriptomes of three different stages of fruit ripening (pre-climacteric, climacteric and post-climacteric) were also analyzed. The analysis of the RNAseqatlas presented here reveals strong differences in gene expression patterns between different organs, especially between root and flower, but also reveals similarities among the gene expression patterns in other organs, such as stem, leaves and aerial buds (vegetative organs) or seed and fruit (storage organs). Important regulators, functional categories, and differentially expressed genes involved in avocado fruit ripening were identified. Additionally, to demonstrate the utility of the avocado gene expression atlas, we investigated the expression patterns of genes implicated in fatty acid metabolism and fruit ripening.

**Conclusions:**

A description of transcriptomic changes occurring during fruit ripening was obtained in Mexican avocado, contributing to a dynamic view of the expression patterns of genes involved in fatty acid biosynthesis and the fruit ripening process.

**Electronic supplementary material:**

The online version of this article (doi:10.1186/s12864-015-1775-y) contains supplementary material, which is available to authorized users.

## Background

Avocado (*Persea americana* Mill.) is a crop plant with oleaginous fruits belonging to the Magnoliidae clade, a basal lineage of flowering plants. It is a member of Lauraceae, a large family of about 50 genera and approximately 2500–3000 species, mostly trees [1, 2]. Despite its recent introduction to international commerce, the avocado is no longer just an exotic fruit; it has been rapidly incorporated as a key component in the diet of many countries [3]. Although Mexico is the world’s largest producer and consumer of avocados (about 28 % of total world production), there are at least ten other countries with annual production of over 100,000 t of avocado fruit (FAOSTAT, 2011;http://faostat.fao.org/DesktopDefault.aspx?PageID=339&lang=es).

*P. americana* comprises no fewer than three well-recognized varieties, geographical ecotypes, or botanical races, also known as horticultural races. The Mexican race, *P. americana* var. *drymifolia* (Mexican avocado), is adapted to the tropical highlands and constitutes the most commonly used rootstock in Mexican orchards; *P. americana* var. *guatemalensis* (L.O. Williams), the Guatemalan race, which grows preferentially at medium elevations in the tropics, and the West-Indian race, *P. americana* var. *americana*, which is typically cultivated in the lowland humid tropics [2]. Commercial avocado production is based on grafting cultivars onto rootstocks of Mexican and Guatemalan races; the cultivars grown in subtropical climates are selections from these races or hybrids of them, with the Guatemalan genotypes being the dominant horticultural race among subtropical avocado cultivars [4]. However, the Mexican genetic background contributes to avocado diversity with a plethora of desirable, selected characteristics in commercial varieties, such as cold tolerance, smaller tree size, high oil content, early maturity, and smooth fruit skin. It is imperative to identify key genes and the signaling pathways associated with these traits, as well as to study the allelic diversity present among botanical races for these desirable traits.

The avocado fruit accumulates oil instead of sugar unlike most fruits, probably as a consequence of co-evolutionary processes developed with ancient neotropical megafauna that became extinct about 30,000–11,000 years ago [5]. Avocado has been described as the most nutritious of all fruits [3], as the mature fruit flesh of avocado contains about 20 % beneficial fatty acids, 6 % carbohydrates, 2 % protein, and vitamin precursors and antioxidants such as carotenoids and vitamins E, C, B2, B12, B1, K and D [6]. The mesocarp of the Mexican race avocado possesses up to 25–30 % oil content, of which nearly 90 % is mono-unsaturated oleic, palmitic and linoleic fatty acids. Although avocado is a strongly climacteric fruit, its ripening or softening process does not take place during maturation on the tree, but instead it starts several days after the fruit has been picked. In the avocado fruit oil content increases in the mesocarp a few weeks after the fruit sets, and healthy fruits on trees continue to grow and accumulate oil for several months after maturation [7]. Once an avocado fruit has been detached from the tree, an ethylene-dependent ripening process is triggered, orchestrating flesh softening, skin color change and lipid biosynthesis. Upon ripening completion, concentrations of unsaturated fatty acids increase and those of saturated fatty acids decrease [8].

The production of essential oils in avocado vegetative organs is influenced by environmental and developmental conditions. However, the distinctive characteristics of the avocado fruit, including secondary metabolite production, are genotype-dependent. For example, the chemical composition of the leaves of the Mexican race is distinctive in its anise scent, which is absent from the two other horticultural races. Estraole, which represents 60 % of the total essential oils in leaves of the Mexican race, is responsible for this trait [9, 10]. Essential oils contain a variety of volatile molecules such as terpenes and terpenoids, phenol-derived aromatic components, and aliphatic compounds; these are widely used for various pharmaceutical, sanitary, cosmetic, agricultural and food purposes, and in nature, they function as herbivore repellants and pollinator attractants [10, 11]. Understanding the biosynthetic pathways of such bioactive compounds by associating functional genomics information with the enzymes involved in the metabolic pathways is fundamental for their commercial production.

Because to date there are only limited avocado genomic resources, carrying out comprehensive gene expression profiling is a challenge requiring large-scale analysis of transcriptomic data. Breakthroughs in next generation sequencing technology and data analysis during the last 10 years have made it possible to generate reference transcriptomes in the absence of a reference genome at a relatively low cost [12]. Reference transcriptomes can be used to perform comparative expression profiling by methods such as digital gene expression profiling [13]. Here, we present the *de novo* assembly of the Mexican avocado (*Persea americana* var. *drymifolia*) transcriptome, based on hybrid sequencing datasets derived from GS-FLX+ Roche and MiSeq Illumina platforms. Additionally, using a high-throughput sequencing platform, we develop a gene expression atlas of the avocado transcriptome in which a total of six different avocado organs and three fruit ripening stages (pre-climacteric, climacteric and post-climacteric) were included. To confirm the utility of the avocado transcriptome atlas, we specifically analyzed the expression of genes involved in acyl-lipid metabolism, ripening processes, and organ-specificity. Our approach generated over 67,000 unigenes with high quality annotations, providing an unprecedented coverage of the avocado transcriptome. The availability of the avocado gene expression atlas should facilitate additional studies on the basic biology of avocado, while also supporting applied research to improve this increasingly important crop.

## Results and discussion

### Sequencing and assembly of the Mexican race avocado transcriptome

**Fig. 1:**
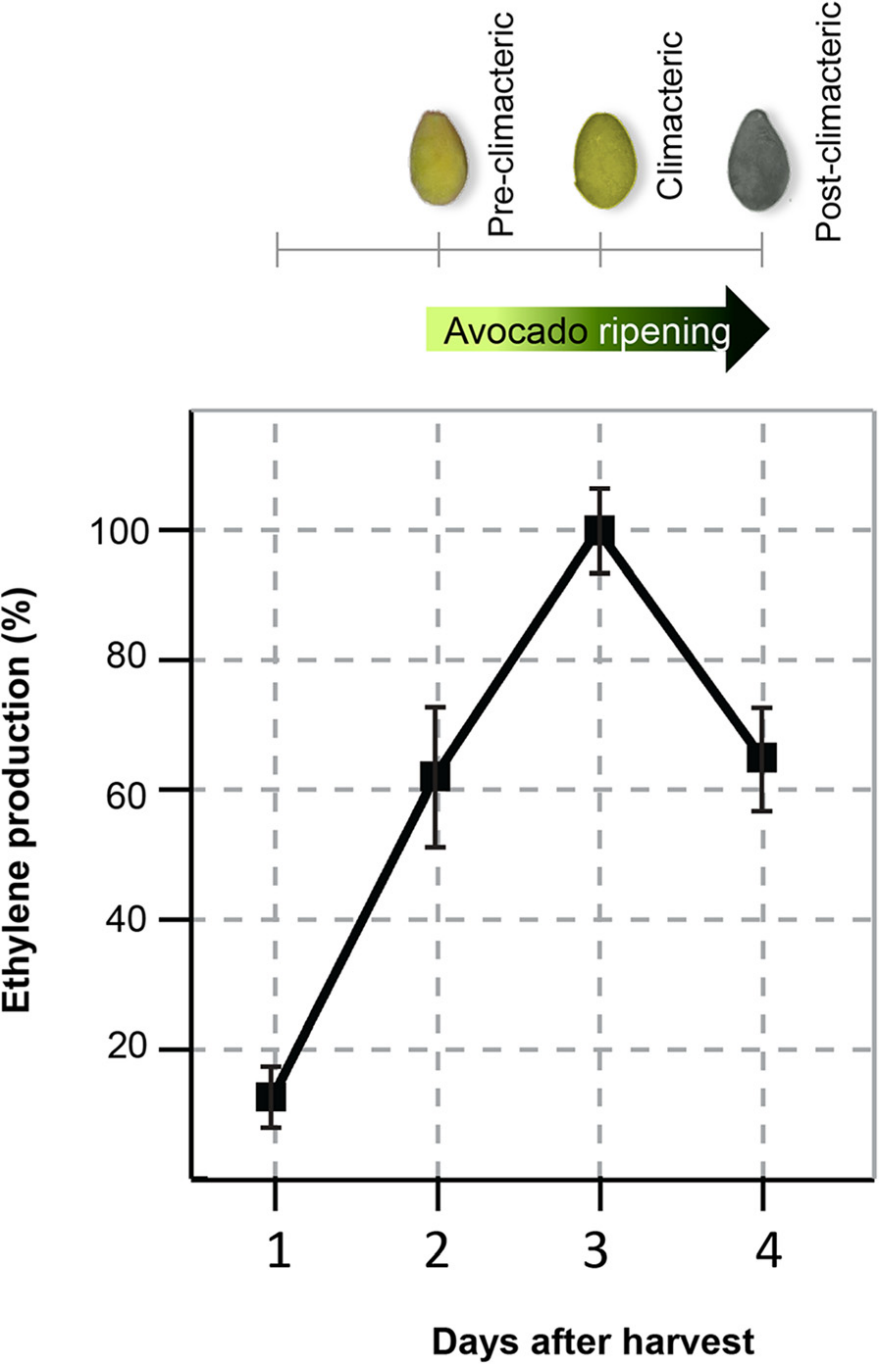
Ethylene production rises during avocado ripening. Ethylene was measured by gas chromatography during the subsequent 4 days after harvest. Maximum ethylene production was adjusted to be 100 %. Three independent biological replicates were analyzed

In order to obtain sequences for as many avocado (*Persea americana* var. *drymifolia*) genes as possible, a cDNA library from an RNA pool isolated from seeds, roots, stems, leaves, aerial buds, flowers and pre-climacteric, climacteric and post-climacteric fruits was generated and sequenced using the GS-FLX+ (Roche) and MiSeq (Illumina) sequencers. MiSeq produced paired-end reads of length 250 bases while GS-FLX+ sequencer generated less reads (unpaired) but the average length was ~3× longer. It is well known that a mixed platform approach (hybrid assembly) can improve the number of full length genes through the inclusion of longer reads, while a higher paired-read coverage increases the detection of low abundance transcripts [14]. Pre-climacteric, climacteric and post-climacteric fruit stages were determined according to their ethylene production (see Methods). It should be noted that unlike other commercial varieties such as ‘Fuerte’, which show the maximum ethylene production seven days after harvest [15], the climacteric physiological stage (which marks the end of fruit maturation and the beginning of fruit senescence) is detected 3 day after harvest in Mexican avocado (Fig. 1).

Reads generated by GS-FLX+ were masked using the SeqClean software pipeline to eliminate sequence regions that would cause incorrect assembly while the MiSeq read pairs (2 × 150 bp) were trimmed and/or merged together using the SeqPrep pipeline (see Methods for more details). To carry out the assembly process, 4,530,278 high quality reads (931,834 generated by GS-FLX+ and 3,598,444 paired-reads generated by MiSeq) were considered (Additional file 1: Table S1).

Many assemblers have been developed to assemble reads generated by Next Generation Sequencing platforms (NGS). The overlap layout algorithm is able to handle the longer reads of GS-FLX+, and programs such as MIRA [16] incorporate it. Trinity [12] in contrast is a de-Bruijn graph-based assembler developed for short reads. We compared the performance of the MIRA v3.4.1 and Trinity assemblers, both previously used in several analyses of plant transcriptomes [17, 18]. For each assembler, we used the default parameters recommended for transcriptome assembly. Standard metrics describing the assembly process, such as number of contigs ≥1 Kb, average contig lengths and maximum contig size were used to compare the assembly programs (Additional file 1: Table S2). Considering that many contigs representative of unique genes are often produced in *de novo* assemblies due to the presence of variant alleles, sequencing errors, and alternative splicing of transcripts, the resulting contigs (derived from both assemblies) were filtered to eliminate redundant sequences and then passed through a second assembly step using the CAP3 assembler [19] (see Methods for more details). A unigene set (83,650 sequences) from *P. americana* was generated including resulting contigs (25,665) and “singlets” (57,985; 64.2 % from MIRA and 33.8 % from Trinity) derived from the CAP3 run with a minimum size of 200 bp (Additional file 2). It should be noted that “singlets” are the contigs generated from the first steps of MIRA or Trinity assemblies that were not reassembled by CAP3. The average length of unigenes was 816.21 bp (ranging from 0.2 to 8.6 kb) (Additional file 1: Table S2). Considering the mean size of coding sequences (≈942.16 bp) in *Amborella trichopoda*, a basal angiosperm species [20, 21], it was expected that a large percentage of these avocado unigene transcripts may represent full-length cDNAs. A comparison of *P. americana* unigenes against the unpublished ca. 800 Mbp draft genome of *P. americana* var. *drymifolia* (unpublished data) using BLASTN (e-value 10^−3^) shows that 94.65 % of the transcripts had a significant hit against the genome (98 % of alignment length and minimal sequence identity of 90 % over the complete alignment).

To annotate the avocado transcriptome, we performed BLASTX alignments (*e*-value of ≤10^−03^ and a bit score ≥25) between the unigene set and several protein databases, including *Arabidopsis thaliana*, *Amborella trichopoda* and plant proteins available in the Reference Sequences (RefSeq) collection of NCBI. We found that 67,709 (80.94 %) unigenes of *P. americana* show high identity to at least one plant protein; the remaining (15,941 unigenes) had no function assigned (Additional file 1: Table S3). In a total of 14,845 avocado unigenes, an individual high-scoring segment pair (HSP) produced by BLASTX covered at least 80 % of the target protein. Results indicated that 34,218 distinct plant proteins could be identified among the 63,459 unigenes that showed significant similarities against RefSeq database (Additional file 1: Table S3). We further compared *P. americana* unigenes against the Pfam (Protein families) domain database (Additional file 1: Table S3; see Methods for more details) [22].

### Functional annotation

**Fig. 2:**
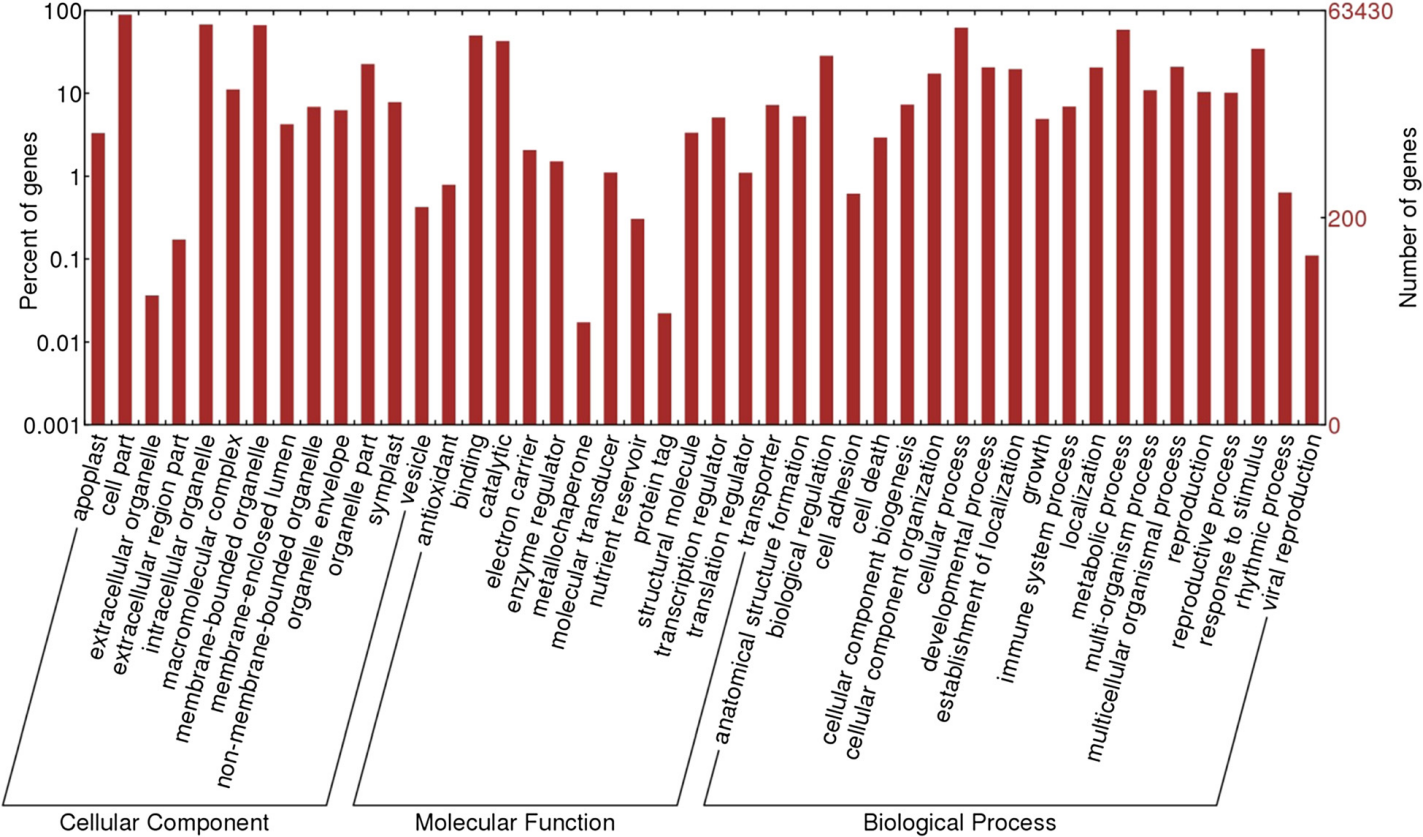
Gene ontology classification of *P. americana* transcriptome. Unigenes with BLASTX matches against the Arabidopsis proteins were classified into three main GO categories (cellular components, molecular functions and biological processes). The *left*-*hand scale* on the y-axis shows the percentage of unigenes belonged to each category. The *right*-*hand scale* on the y-axis indicates the number of unigenes in the same category

**Fig. 3:**
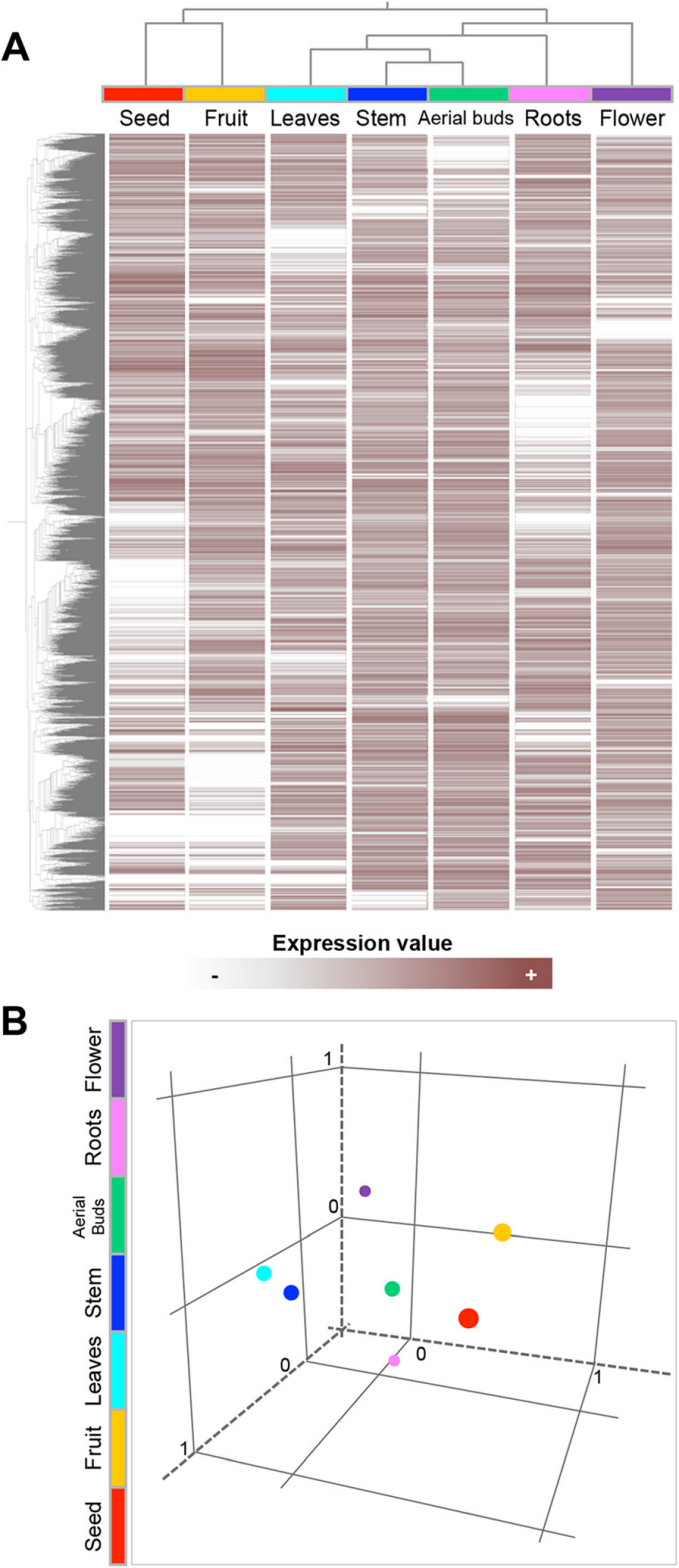
Profiling expression of *P. americana* transcriptome. **a** Hierarchical clustering shows expression levels of unigenes across different avocado organs. **b** Principal component analysis [seed (*red*), fruit (*orange*), leaves (*cyan*), stem (*blue*), aerial buds (*green*), roots (*pink*) and flower (*purple*)]

The results of BLASTX searches against the *Arabidopsis thaliana* protein database were used for gene ontology (GO) mapping and annotation. Based on the Arabidopsis top hits, we obtained the GO annotations for the avocado unigenes, and WEGO software [23] was used to perform GO functional classification into the three major classes (Fig. 2; Additional file 1: Table S3). Among the unigenes with Arabidopsis hits, 63,430 (75.82 %) were assigned to gene ontology classes with 547,032 functional terms. Biological processes comprised the majority of the functional terms (259,327; 47.40 %), followed by cellular component (151,379; 27.67 %) and molecular functions (136,326; 24.92 %). Within the biological processes category, cellular (39,365 unigenes) and metabolic (37,208 unigenes) processes were prominently represented. To further predict the metabolic pathway in *P. americana*, the assembled unigenes were annotated with corresponding enzyme commission (EC) numbers in the KEGG automatic annotation server (KAAS; [24]) using *Arabidopsis thaliana* and *Oryza sativa* as references (Additional file 1: Table S3). A total of 2559 unigenes were mapped to 202 pathways corresponding to five KEGG modules: energy metabolism, carbohydrate and lipid metabolism, nucleotide and amino acid metabolism, genetic information processing, and environmental information processing. Additionally, the modules energy metabolism (structural complex) and metabolism (functional set) were also identified (Additional file 3: Figure S1 and Additional file 4: Table S4). Ribosome had the largest number of unigenes (78 members, M00177), followed by glycolysis (Embden-Meyerhof pathway; 62 members, M00001), reductive pentose phosphate cycle (Calvin cycle; 47 members, M00165), gluconeogenesis (40 members, M00003), and spliceosome (30 members, M00354) (Fig. 3; Additional file 4: Table S4).

### Expression map of the *P. americana* unigenes (the transcriptome atlas)

In the past, gene expression atlases from different plant species have been established by using massive parallel-signature sequencing and array-hybridization technologies [25–30]. Plant transcriptomes strongly vary from one tissue to another [25, 29], and it has been suggested that these variations are responsible, at least in part, for the identities of different plant organs [26]. We used the SOLiD v4.0-sequencing platform to quantify the expression of *P. americana* unigenes in seven different organs: seeds, roots, stem, leaves, aerial buds, flowers and fruits. Fruit was considered as the total of reads generated from pre-climacteric, climacteric and post-climacteric libraries. The resulting reads from these three libraries were also independently mapped. Between 4.18 and 6.84 million SOLiD reads were generated for each of the seven organ libraries; among them, 51.13 % were mapped to the avocado transcriptome (Additional file 4: Table S5). An expression profile matrix containing the unigenes (rows) and the number of mapped reads in each normalized organ-specific transcriptome (columns), was created. To allow for data comparison among samples, a normalization of the reads per Kb per million (RPKM) was performed. A threshold of RPKM ≥5 was used to distinguish expressed genes in at least one of the organs sampled from background [31] (Additional file 4: Table S6). We further investigated how the organs can be classified according to transcript levels using a principal component analysis (PCA) and hierarchical clustering (HC), by employing the Pearson metric on average expression levels for each organ (Fig. 3a and b).

For many of the organ types, the hierarchical plot and the PCA reflect the known similarity of biological functions among organs. For example, transcripts from leaves and stems clustered as a neighboring group, reflecting their physiological similarity (vegetative organs). Likewise, the storage organ transcriptomes (fruit and seed) were grouped together, whereas gene products from roots clustered separately from the rest (Fig. 3b). To assess the relative abundance of gene transcripts among the organ-specific transcriptomes, we used the log-likelihood ratio statistic, *R* [32], which scores reads by departures from the null hypothesis of equal counts in each library given the total number of reads sampled from each library. Higher *R*-values indicate a greater probability of differential expression, whereas *R*-values near zero represent constitutive expression (ubiquitous unigenes; see Methods). By considering as preferentially expressed genes the unigenes with *R*-values ≥15 (true positive rate of ~98 %), a total of 9357 *P. americana* unigenes were selected as preferentially expressed in at least one of the organ analyzed (Additional file 4: Table S7). Of these, 1968 unigenes (224 from seed, 192 from leaves, 22 from stem, 503 from roots, 56 from aerial buds, 654 from flowers and 313 from fruit) could be considered as organ-specific genes because the reads were derived from a single library (Additional file 4: Table S8). Although 33.63 % of organ-specific unigenes corresponded to sequences that could not be annotated, in the remaining unigenes, these organ-specific expression data are consistent with previously documented expression patterns of known genes. For example, the avocado unigene UN06501, a homolog of the Arabidopsis high-affinity phosphate transporter PHT1-3 gene (AT5G43360), is expressed in roots [33]. Four well-characterized floral pattern determination genes, AGL2 (AT5G15800; homologous to UN50397), AGL6 (AT2G45650; homologous to UN22054), AP3 (AT3G54340; homologous to UN19620) and PI (AT5G20240; homologous to UN19579), exhibited specific expression patterns among floral organs [34–37]. Likewise, in leaves, several avocado unigenes homologous to Arabidopsis light-harvesting chlorophyll a/b (LHCB) proteins were expressed in an organ-specific manner. This was expected, since in higher plants and algae, LHCB proteins are major components of the light-harvesting complex of photosystem II (PSII) in chloroplasts, which is responsible for light harvesting and energy transfer to reaction centers [38, 39]. Moreover, the fruit-specific avocado unigene UN44288 is homologous to AGL4 (AT3G02310), a transcription factor that has been recently characterized for its main role in apple fruit development and ripening [40]. Interestingly, an oleosin-encoding gene (UN35609) homologous to AT3G18570 showed a higher expression level in fruits than in seeds (Additional file 4: Table S7). Oleosins have been described as key structural and signaling components of seed oil bodies that form during the desiccation process and prevent coalescence of the oil [41]. This suggests that oleosins could be involved in avocado fruit development, beyond their known functions in seeds. In addition to its high lipid content, the avocado fruit contain several bioactive phytochemicals, including carotenoids [42]. The homolog of carotenoid biosynthesis-associated epoxycarotenoid dioxygenase NCED3 (AT3G14440), unigene UN43474, was highly expressed in the fruit transcriptome (Additional file 4: Table S7). Seven different avocado unigenes (UN26753, UN45556, UN14598, UN31059, UN39923, UN51437 and UN56049), among the highest expression levels from seed-specific genes, are homologs of PAP85 (AT3G22640), a member of the large superfamily of cupins, which are expressed during seed development and act as a nutrient reservoir [43]. The homolog to fasciclin-like arabinogalactan protein FLA12 (AT5G60490; unigene UN17820) exhibited one of the highest expression levels of stem-specific genes, which is consistent with previous reports showing that expression of some members of the FLA gene family is correlated with the onset of secondary-wall cellulose synthesis in Arabidopsis stems and with wood formation in the stems and branches of trees, suggesting a biological role in avocado stem development [44]. Together, these results indicate that the transcriptome atlas of *P. americana* presented here provides an accurate estimation of organ-specific gene expression patterns that may assist functional interpretations.

### Transcriptome changes during avocado fruit development and ripening

We identified unigenes that are differentially expressed during fruit development and ripening using a similar approach to that described above (RPKM values derived from mapped reads and a threshold of *R* ≥15 to select differentially expressed genes; see Methods). First, the RPKM values between flower and fruit were compared in order to identify some genes that may play important roles during fruit development. A total of 382 unigenes were selected due to the significant increase of their transcripts in the fruit/flower comparison. We surveyed some of these genes in order to determine their potential roles during fruit development (Additional file 4: Table S9). A total of 10 avocado unigenes (UN54812, UN37501, UN61103, UN59855, UN53293, UN48116, UN58333, UN64333, UN64163 and UN68295), homologs of Arabidopsis metallothionein MT2A (AT3G09390), were identified. Metallothioneins are small cysteine-rich proteins required for heavy metal tolerance in animals and fungi. In plants, metallothionein genes are up-regulated in response to heavy metal stress [45] but also participate in natural and induced leaf senescence [46, 47], ethylene-induced abscission [48] and biotic and abiotic stress responses [49–51]. However, metallothioneins have been also identified as up-regulated genes during climacteric fruit development in banana [52], apple [53], and kiwifruit [54] and in non-climacteric fruit such as grape [55], *Citrus unshiu* [56], strawberry [57] and pineapple [58]. Despite their abundance, the function of metallothioneins during fruit development still remains largely unknown. Chitinases were also found to be a well-represented group of fruit specific unigenes (18 in total), most of them homologs of the basic chitinase CHIB/PR-3 (AT3G12500). Consistently, besides the role of chitinases in plant defense [59], these proteins have also been associated with fruit development in both climacteric [60] and non-climacteric fruits [61]. Pectate lyases constitute an additional protein family that has been suggested to play an important role in fruit ripening and softening [62]. This is consistent with the fact that eight avocado unigenes, homologous to four different members of the Arabidopsis pectin lyase-like superfamily (AT1G80170, AT3G07850, AT3G07970 and AT5G18990), showed significant increase in the frequency of transcriptional units in the flower/fruit comparison.

**Fig. 4:**
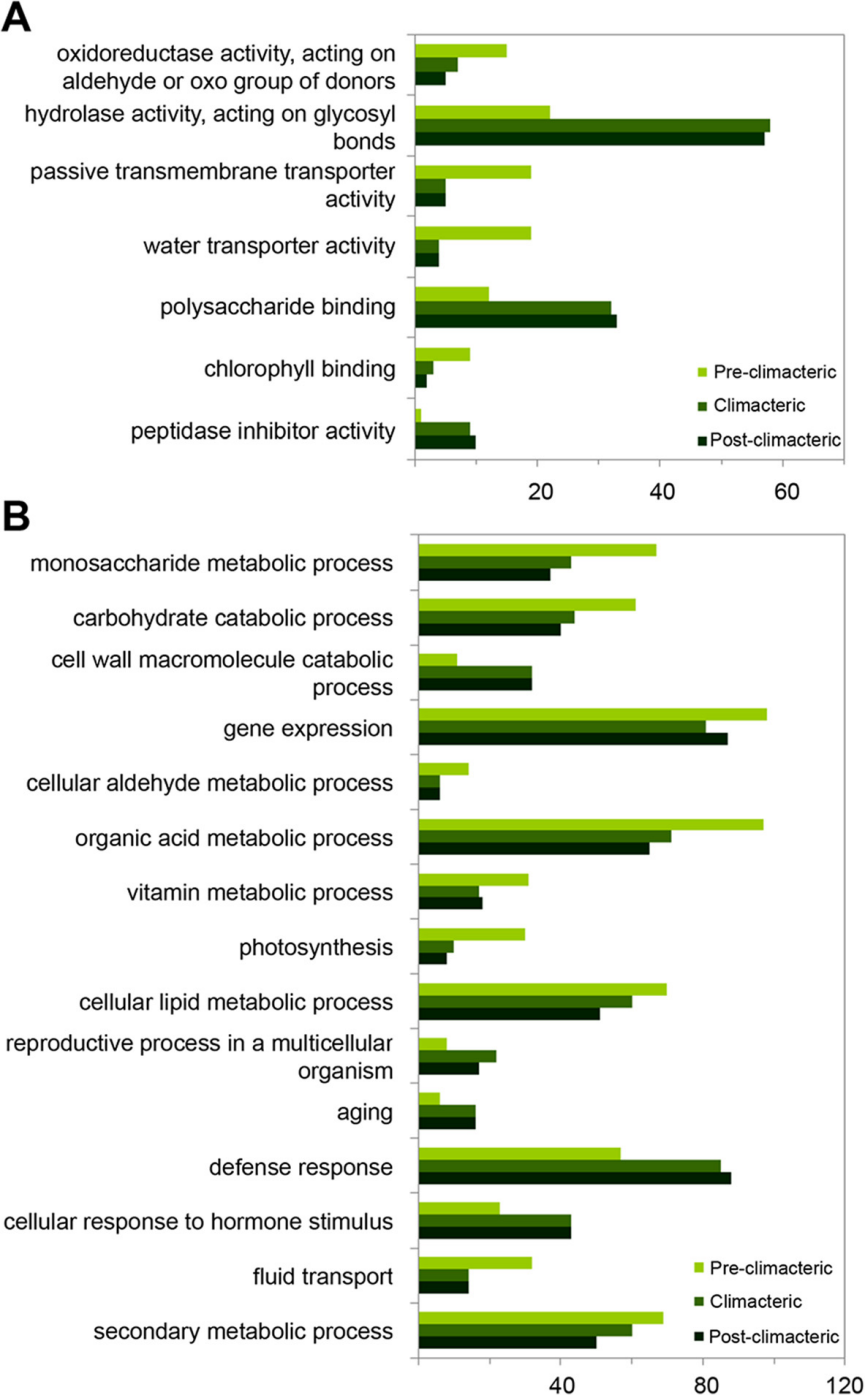
Functional categorization of differentially expressed genes during avocado fruit ripening. Genes were categorized based on GO annotation, and the number for each category is displayed based on molecular function (**a**), or biological process (**b**). Data presented represent GO terms at level 4

One-thousand-two-hundred-thirty unigenes out of 16,025 (RPKM values ≥5) were identified as differentially expressed during the ripening process of the avocado fruit (Additional file 4: Table S10). In order to find over-representation of a given function, GO categories were assigned to the unigenes differentially expressed in each ripening stage (pre-climacteric: 756 unigenes, climacteric: 841 unigenes, and post-climacteric: 812 unigenes). Only GO sub-categories that showed significant differences in the ‘molecular function’ and ‘biological process’ categories were analyzed. Figure 4a shows the differentially expressed unigenes within the ‘molecular function’ category. Sub-categories ‘Hydrolase activity, acting on glycosyl bonds’, ‘polysaccharide binding’ and ‘peptidase inhibitor activity’ showed a significant increase in the number of genes while ‘oxidoreductase activity, acting on the aldehyde or oxo group of donors’, ‘passive transmembrane transporter activity’, ‘water transporter activity’ and ‘chlorophyll binding’ showed a decrease. Regarding ‘biological process’ GO terms, the majority of differentially expressed unigenes appeared to be related to some major biological changes, including ‘monosaccharide metabolic process’, ‘carbohydrate catabolic process’, ‘cell wall macromolecule catabolic process’, ‘gene expression’, ‘cellular aldehyde metabolic process’, ‘organic acid metabolic process’, ‘vitamin metabolic process’, ‘photosynthesis’, ‘cellular lipid metabolic process’, ‘reproductive process in a multicellular organism’, ‘aging’, ‘defense response’, ‘cellular response to hormone stimulus’, ‘fluid transport’, and ‘secondary metabolic process’ (Fig. 4b). The complete list of GO categories is provided in Additional file 4: Table S11. These results are consistent with the notion that the principal changes associated with ripening include color (loss of green color and increase in non-photosynthetic pigments that vary depending on species), firmness (softening by cell wall degrading activities and alterations in cuticle properties), taste (increase in sugar and decline in organic acids), and flavor (production of volatile compounds providing characteristic aromas) [63].

**Fig. 5:**
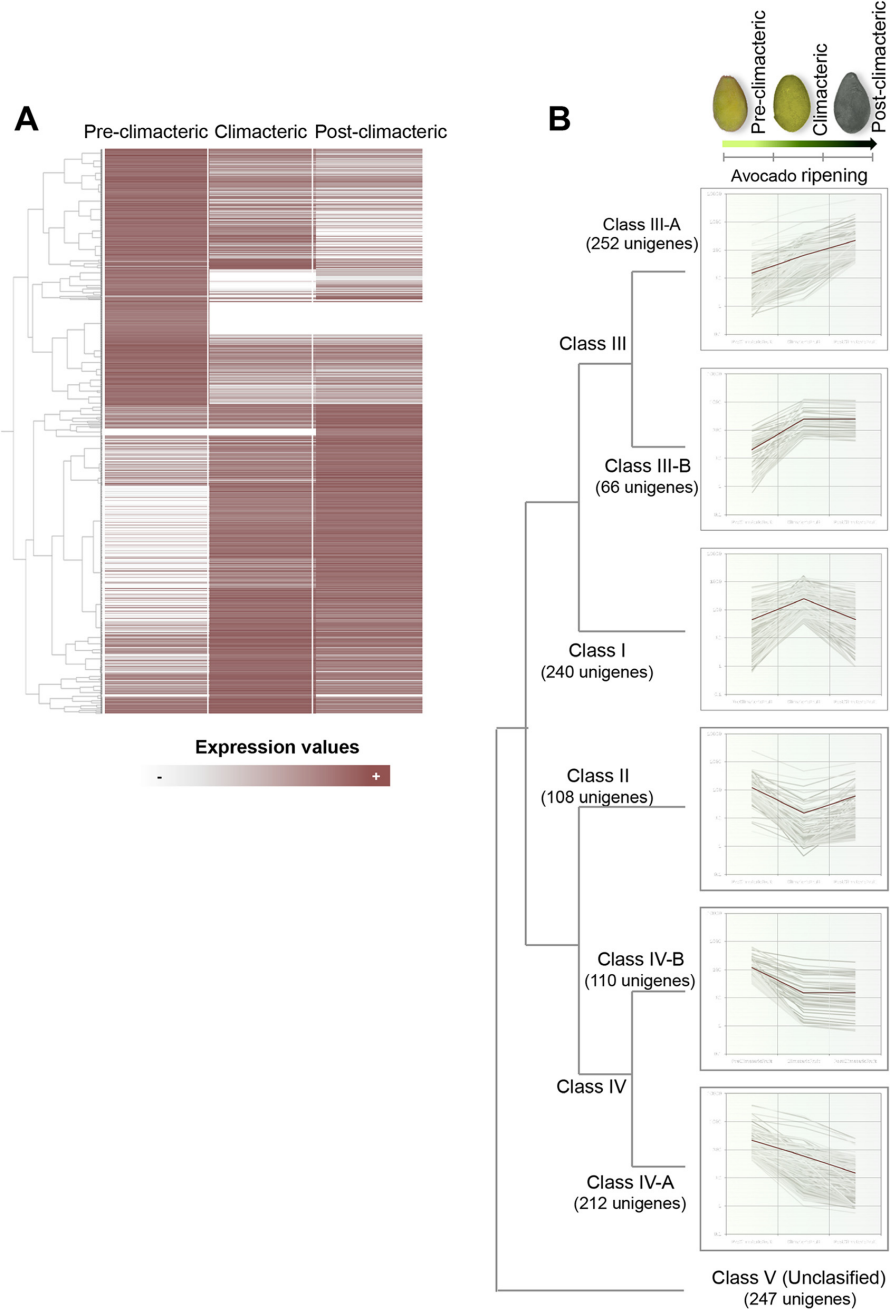
RNA-seq based transcriptome dynamics of avocado during fruit ripening. **a** The log2 of RPKM values for each gene was used for the hierarchical clustering analysis at each of the three selected ripening stages (pre-climacteric, climacteric and post-climacteric). **b** The 1235 differentially expressed unigenes were classified into 5 regulation patterns (classes I-V, respectively). Classes III and IV were subdivided into two sub-classes each. The graph shows the expression profile of unigenes for each class. *Gray lines*, expression profiles for individual unigenes. *Brown lines* represent the average intensities of unigene members of the clusters. For additional information, see Additional file 4: Table S10

Differentially expressed unigenes during fruit ripening were also examined by cluster analysis of gene expression patterns, which arranged the gene products into 5 major groups named as classes I-V (Fig. 5a and b; Additional file 4: Table S10). The most represented classes comprised the unigenes whose expression increased (class III) or decreased (class IV) during fruit ripening. Both classes were subdivided into two sub-classes (A and B, respectively). Classes III-A and IV-A comprised the unigenes whose expression increased or decreased continuously during fruit ripening, while classes III-B and IV-B showed increase or decrease at the climacteric stage, with similar expression levels maintained at post-climacteric stages. Classes I and II represent the unigenes in which the highest or lowest expression levels were detected at the climacteric stage. Finally, class V represents the remaining unigenes that were not grouped into none of the classes mentioned above. Differential gene expression patterns observed in the RNA-seq experiments were validated by real-time PCR for eight avocado unigenes differentially expressed during fruit ripening (Additional file 3: Figure S2). These genes showed the same expression pattern in both RNA-seq (measured as RPKM values) and in the real-time PCR analyses.

### Genes relevant to avocado fruit ripening

In ripening of climacteric fruits, the expression of some members of the gene families encoding ACC synthase and ACC oxidase is induced, regulating the biosynthesis of ethylene [64]. Two differentially expressed unigenes (UN03798 and UN39755) were found to be homologs of Arabidopsis ACC synthase (AT3G61510), and one, UN38306, a homolog of ACC oxidase (AT1G62380). In relation to their expression profiles, the unigenes homologous to the ACC synthase were classified into class III-A, while the homolog of ACC oxidase was classified into class II (Fig. 5b). Therefore, during avocado fruit ripening, the ethylene burst seems to be regulated mainly by ACC oxidase. This is consistent with the notion that in climacteric fruits the rate of ethylene production is well correlated with the pattern of accumulation of ACC synthase and ACC oxidase gene transcripts [65]. Avocado unigenes homologous to ethylene response factor ERF110 (AT5G50080; UN29560 and UN42714) and ethylene receptor EIN4 (AT3G04580; UN01530 and UN24061), which play key roles in the signaling of ethylene responses [66], were also identified as members of class III-A (Fig. 5b).

The genetic and physiological characterization of tomato ripening mutants, *ripening*-*inhibitor* (*rin*; [67]), *non*-*ripening* (*nor*; [68]) and *colorless non*-*ripening* (*cnr*; [69]), together with the molecular characterization of the mutated genes, have demonstrated that several important regulatory factors must be properly coordinated with the ethylene signal to properly activate and orchestrate the ripening program. RIN [70], NOR [71] and CNR [72] genes encode transcription factors belonging to the MADS-box, NAC-domain, and SBP-box families, respectively, that act upstream of ethylene biosynthesis and perform key functions in the control of fruit ripening [73]. RIN [74] and two regulatory proteins more recently identify as involved in fruit ripening, the TAGL1 MADS-box factor [75] and the HB-1 homeobox protein [76], are able to bind to the promoter region of ACS2 [74] and ACO1 [76] genes, respectively, demonstrating that transcription factors directly regulate the activity of ethylene biosynthesis genes in tomato. A bi-directional best BLAST hit approach was used to identify the avocado orthologs of tomato genes involved in ripening (see above). Using the SeaView program [77] the protein-coding nucleotide sequences were then aligned based on their corresponding amino acid translations to calculate the percent identity at nucleotide and amino acid levels (Additional file 5). The identities of avocado/tomato genes ranged from 48.82 to 63.53 % (Additional file 4: Table S12) and with only one exception (HB-1; UN22151), their expression levels were considerably higher in flowers than in the remaining organs sampled. Meanwhile, NOR and HB-1 avocado transcripts increased during fruit ripening while CNR decreased. RIN and TAGL1 showed very low transcript levels (RPKM <5) only at the preclimacteric stage (Additional file 3: Figure S3). Avocado is considered a basal angiosperms with origin near the split between monocot and eudicot plant species. The finding that some but not all transcription factors involved in tomato fruit ripening are up-regulated or even expressed during avocado fruit ripening suggests that part but not all of the transcription factor wiring was ancestrally present and that substantial rewiring occurred during the evolution of modern eudicots.

It has been reported that a thaumatin-like protein and a class I chitinase are some of the most abundant ripening-associated proteins in banana fruit [60]. Genes encoding these proteins were also found to have differential expression during ripening in avocado. Avocado unigene UN10274, a homolog of a pathogenesis-related thaumatin (AT1G20030), clustered into class III-A (transcripts continuously rising; Fig. 5b), the same as several unigenes (16 in total; see Additional file 4: Table S10) homologous to PR-3 (AT3G12500), a well-known class I chitinase strongly induced when plants respond to wounding or infection by fungal, bacterial, or viral pathogens [78]. Additional unigenes (UN43717 and UN45051) with different expression profiles (class I) were identified as homologs of AT4G01700 and AT2G43610, class II and IV chitinases, respectively. Therefore, comparisons with data reported for other climacteric fruits (including ethylene responsive genes) facilitated identification of putative conserved orthologous ripening-related genes, which serve as an initial set of candidates for assessing the conservation of gene activity during the evolution of fleshy-fruited plant species.

### Acyl lipid metabolism of *Persea americana* fruits

**Fig. 6:**
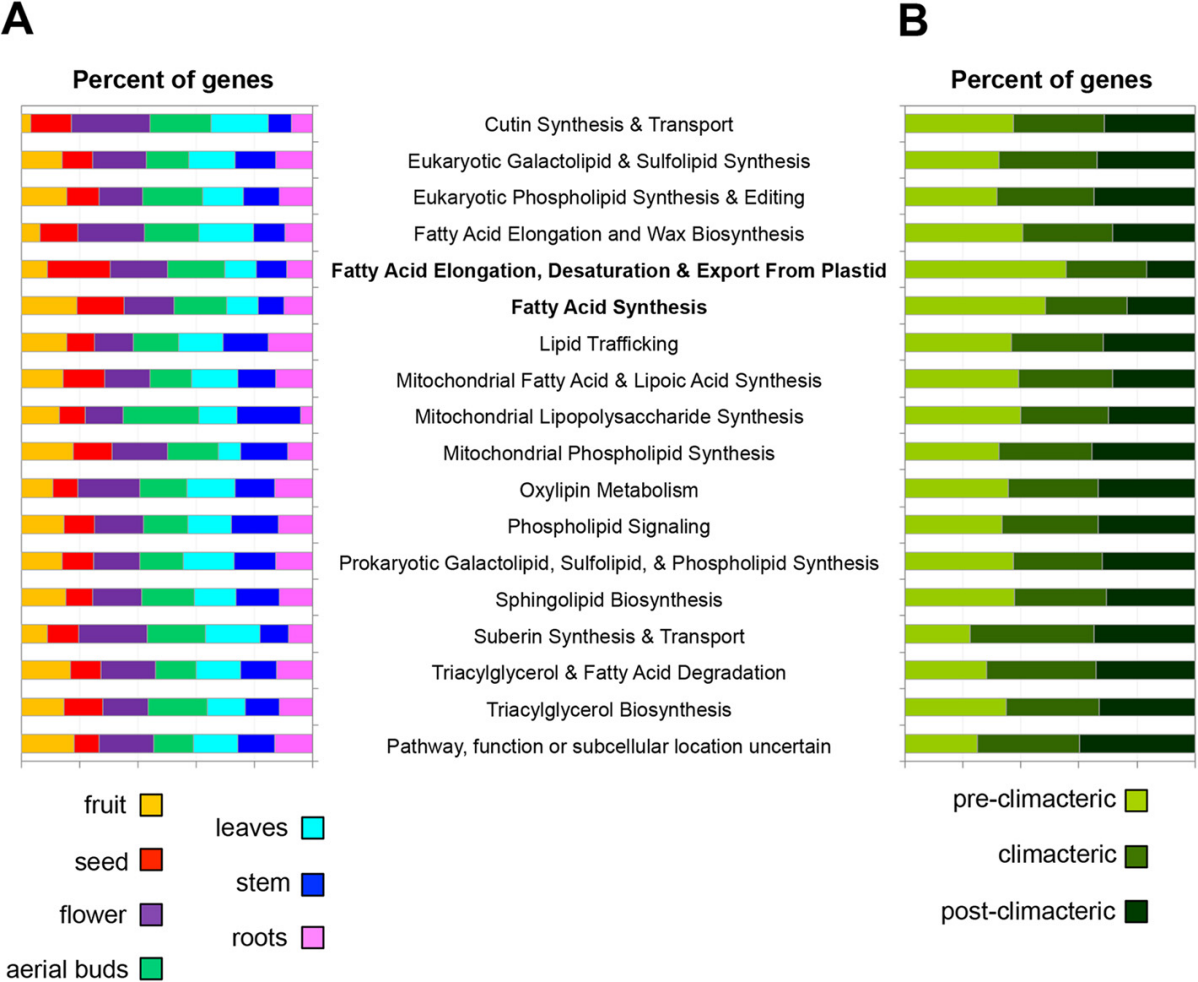
Totals of *P. americana* unigenes suspected to be involved in acyl-lipid metabolism. Unigenes were annotated with a translated BLAST (*e*-value ≤10^−03^ and a bit score ≥25) against Arabidopsis proteins and further annotated based on information at the Arabidopsis Lipid Gene Database (http://aralip.plantbiology.msu.edu/). For additional information, see Additional file 4: Table S11. **a** Percent of the genes detected in each of the organs sampled and (**b**) during fruit ripening

Previously, an acyl lipid metabolism gene database was developed for Arabidopsis [79], and this was recently expanded by deep transcriptional profiling of developing seeds from four different oilseed species [80]. This significant effort resulted in detailed sequence information for over 740 genes encoding proteins involved in lipid metabolism, providing a useful resource for construction of additional databases of genes related to lipid biosynthesis from other oilseed or plant species with lipid-rich fruits (http://aralip.plantbiology.msu.edu/). After comparing our assembled transcriptome against the aforementioned database, we found that 1177 avocado unigenes had been annotated as homologous to lipid metabolism genes with transcriptional evidence for their expression (RPKM values ≥5) in at least one of the organ sampled (flower, leaves, roots, stem, aerial buds, seed and fruit) (Fig. 6; Additional file 6). A similar number of unigenes related to the ‘fatty acid synthesis’ category were detected in fruit, seed, flower and aerial bud organs, whereas stem, leaves, and root organs had a lower number of expressed unigenes annotated in the same category. The category ‘fatty acid elongation, desaturation & export from plastid’ shares a similar pattern (Fig. 6a). Interestingly, the number of genes detected in both categories decreases during fruit ripening (Fig. 6b). This was expected since after flowering and during avocado fruit development, the moisture content decreases while the lipid content steadily increases [81], and after the picking, and during the post-harvest ripening period, the avocado fruit does not show significant changes in the fatty acid composition [82]. Our data suggests that the initiation of fruit ripening marks the end of oil accumulation and fatty acid composition.

**Fig. 7:**
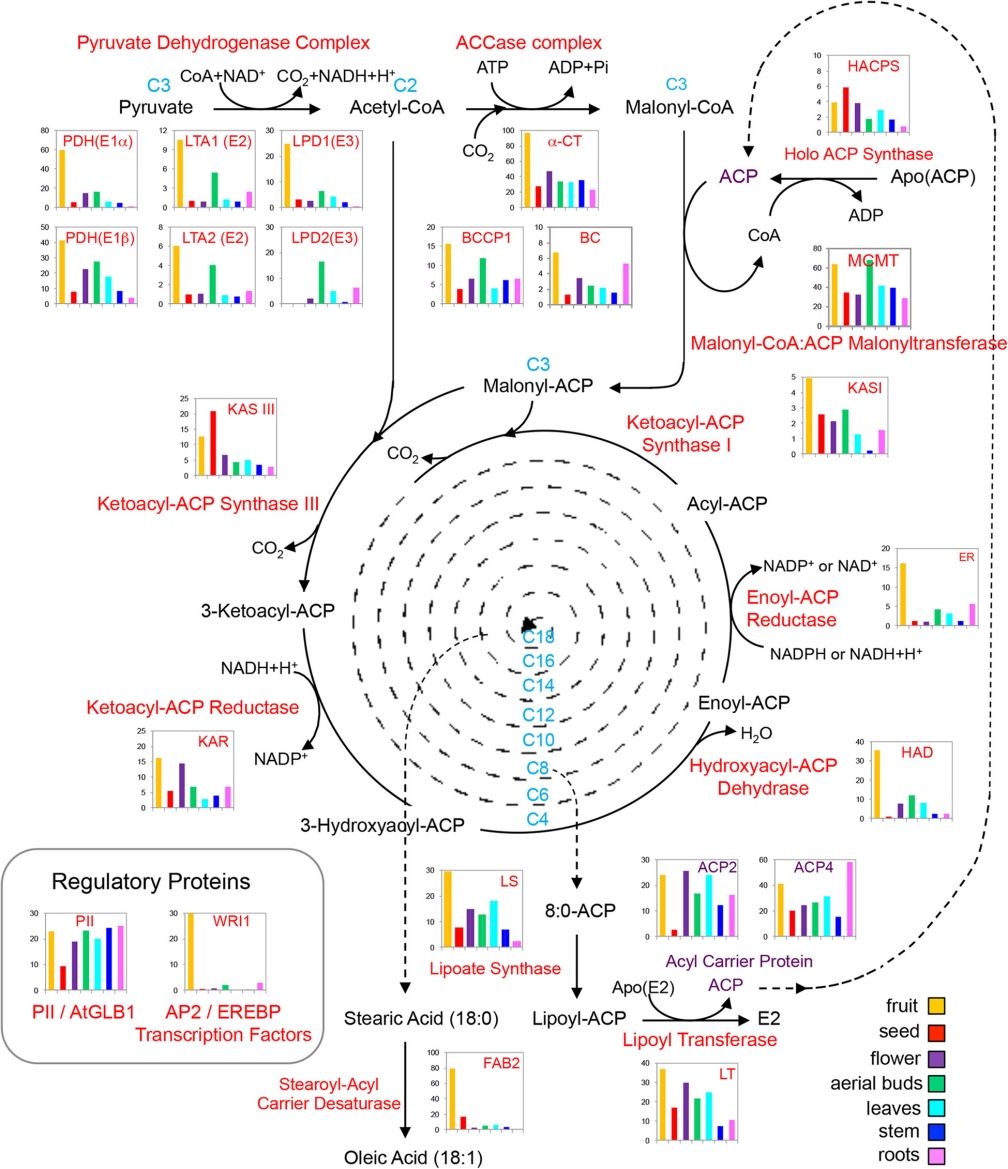
Expression profile of *P. americana* unigenes involved in the fatty acid biosynthetic pathway. The *bar graphs* show the frequency of transcriptional units as the average of RPKM values of all unigenes annotated as homologous to each Arabidopsis gene (represented by *red letters* in the figure). Each avocado organ analyzed is represented by different color: fruit (*yellow*), seed (*red*), flower (*purple*), aerial buds (*green*), leaves (*cyan*), stem (*blue*) and roots (*pink*). This figure was modified from the Arabidopsis Lipid Gene Database (http://aralip.plantbiology.msu.edu/)

**Fig. 8:**
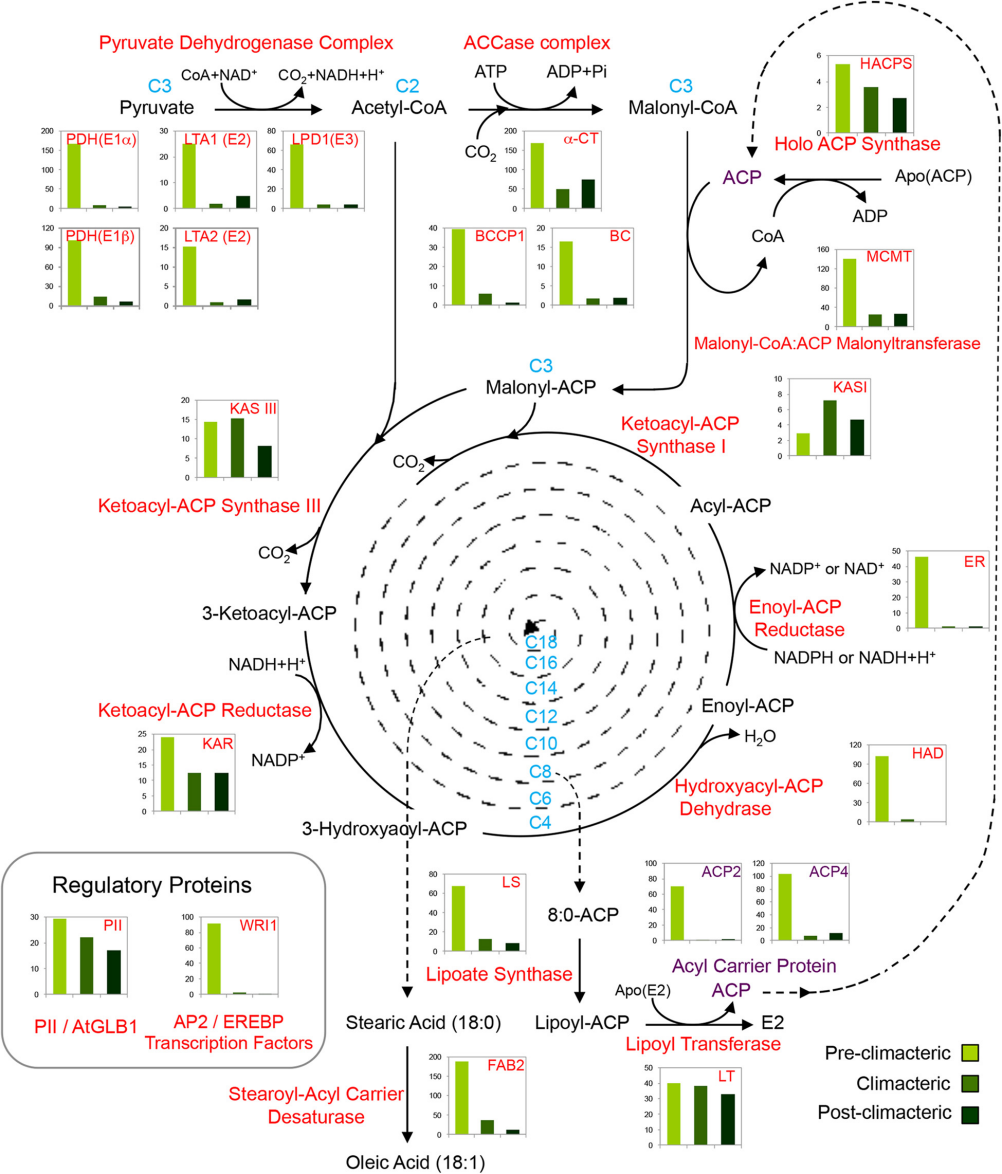
Expression profile of genes coding for fatty acid biosynthesis during avocado fruit-ripening. The *bar graphs* show the frequency of transcriptional units as the average of RPKM values of all unigenes annotated as homologous to each Arabidopsis gene (represented by *red letters* in the figure). Each avocado fruit ripening stage is represented by different color: pre-climacteric (*light green*), climacteric (*green*) and post-climacteric (*dark green*). This figure was modified from the Arabidopsis Lipid Gene Database (http://aralip.plantbiology.msu.edu/)

The average RPKM values of unigenes annotated as homologous to fatty acid biosynthesis genes were calculated in order to estimate expression profiles of their corresponding avocado genes (Additional file 4: Table S13). The transcription levels of the majority of genes involved in fatty acid biosynthesis are significantly higher in fruit than in all the other organs analyzed (Fig. 7); nonetheless, they decrease during fruit ripening (Fig. 8). In addition, according to their expression profiles, three homologs (UN06747, UN21149 and UN33083) of FAB2/SSI2 (AT2G43710) were identified as class IV-A differentially expressed unigenes. FAB2/SSI2, a soluble stearoyl-acyl carrier protein desaturase, is a major enzyme responsible for converting saturated stearic acid (C_18:0_) to monounsaturated oleic acid (C_18:1_) in chloroplasts [83]. Our data are also in agreement with the transcriptome of developing mesocarp of ‘Hass’ avocado that was generated in parallel to these studies where genes involved in acyl lipid metabolism were exclusively investigated [84]. Together, these results suggest that lipid accumulation and changes in fatty acid composition (for example, some fatty acid desaturations) occur during avocado fruit development, and probably both processes stop a few days after the fruit has been harvested (at the pre-climacteric stage). The noticeable decrease of the FAB2/SSI2 transcripts, as well as genes involved in fatty acid biosynthesis during fruit ripening, could explain the fact that no significant changes have been detected in avocado fatty acid composition during the post-harvest ripening period [7]. Considering that ethylene production starts at the pre-climacteric stage and quickly increases towards the climacteric state, it is tempting to hypothesize that in avocado fruit, ethylene is perhaps a signaling molecule that once perceived, halts lipid biosynthesis and programs future changes in fatty acid composition.

Although in most avocado varieties over half of the total fat present is in the form of oleic acid (C_18:1_), recent studies have shown that at least 22 different fatty acids can be detected in pulp and seed avocado oils [85]. Palmitic (C_16:0_) and linoleic (C_18:2_) fatty acids are the second major constituents of fruit oils while some others like stearic acid (C_18:0_) are present in trace amounts [85]. The benefits to nutrition and health of avocado fatty acids have been recently reviewed [86]). In addition, avocado acetogenins, such as persin [(+)-(Z,Z)-1-(acetyloxy)-2-hydroxy-12,15-heneicosadien-4-one], a deoxy-derivative of glyceride with close structural homology to the monoglyceride of linoleic acid, has been proposed as an alternative therapy against breast cancer due its necrotic effect in the mammary gland [87]. Considering the importance of avocado to nutrition and health, transcripts encoding enzymes of the biosynthetic pathway from palmitic to linoleic acid, passing through stearic and oleic acids, were reconstructed similarly to described above (Additional file 4: Table S13; Additional file 3: Figure S4). It is worth noting that genes involved in the biosynthesis of linoleic acid (downstream of oleic acid) showed lower expression levels in fruit than in seed. This is consistent with previous reports in which linoleic acid seems to accumulate in greater amounts (around 4-fold, [85]) in seeds than in fruits.

## Conclusion

The avocado transcriptome reported in this study provides a foundation for the molecular genetics and functional genomics required to study the basis of the genetic diversity that determines the different levels and quality of oil accumulation in the fruit of different avocado cultivars, as well as other important agronomic traits for this increasingly important crop. Transcriptomic data will also facilitate the identification of the pathways involved in production of a wide variety of essential nutrients and phytochemicals beneficial for human health that are produced by the avocado. This data will also facilitate the study of early plant evolution since avocado is phylogenetically placed near the separation between monocot and eudicot plants.

## Methods

### Plant material

Avocado (*Persea americana* var. *drymifolia*) samples were obtained from the germplasm bank of the “Instituto Nacional de Investigaciones Forestales y Agropecuarias (INIFAP)” in Uruapan, Michoacán, México. Organs sampled [Leaves: mixed development stages of expanded leaves; Stem: segments from young branches, Aerial buds: developing buds emerging from shoot apical meristem; Flower: whole inflorescences with flowers at multiple stages of development (young, immature, and mature); Seed: isolated from harvested fruits at the mature-green state (approximately 8 months-old); roots: whole root system recovered from in vitro-propagated seedlings [88], or from cuttings grown in pots in a greenhouse]. Fruits in the mature-green state were also harvested for the ripening experiments. Seeds were removed once the fruit was frozen at each rippening stage. All organs/tissues sampled were obtained from a single reference tree (accession 001-01, which has been sequenced as part of the avocado whole-genome sequencing project).

### Characterization of the climacteric behavior of avocado fruit

In avocado and other climacteric fruits such as apple, melon and banana, an ethylene burst is required for normal fruit ripening (reviewed in [89]). According to the method described by Hoffman and Yang [15], ethylene production of each fruit was monitored individually by gas chromatography during the course of ripening until they were sacrificed for RNA extraction.

### RNA sequencing

Total RNA was isolated using the Trizol reagment (Invitrogen) and re-purified with the RNeasy kit (Qiagen) following the manufacturer’s instructions. Five micrograms of total RNA from each sample were pooled to produce sequencing libraries. Additionally, according to ethylene quantification (Additional file 3: Figure S1), three independent extractions were prepared from 8 months-old fruits (pericarp), at the second, third and fourth day after harvest (pre-climacteric, climacteric and post-climacteric stages respectively), allowing us to contrast changes in the transcriptome profile of the avocado fruit at different ripening stages. cDNA preparation, library construction, and sequencing were performed according to Illumina and Roche manufacturer instructions. From the RNA pool, two sequencing libraries were prepared and a single sequencing run was carried out on the GS-FLX+ (Roche) sequencer, and another on the Illumina MiSeq platform. A total of 4,674,756 reads were generated (1,055,903 reads from GS-FLX+ with an estimated average size of 462.27 bp and 3,618,853 paired reads, 2 × 150 bp, from MiSeq). Additionally, using the SOLiD system and the SOLiD RNA *Barcoding* Kit (v4 chemistry), 1/4 run, in which all RNA samples were independently represented using barcodes, was performed. A total of 70,650,328 short reads (up to 50 pb) were generated (Additional file 1: Table S1). Files containing sequence reads and quality scores were deposited in the Short Read Archive of the National Center for Biotechnology Information (NCBI) [Accession number SRS923862].

### Assembly and sequence analysis

To carry out the assembly process only the reads generated with GS-FLX+ and Illumina-MiSeq sequencer were considered. Using the CDHIT program [90], natural and artificial duplicate reads were removed from the data set generated by the GS-FLX+ sequencer. Additionally, reads with an average quality less than 20 (phred score), were also removed. On the other hand, forward and reverse read pairs (generated by Illumina-MiSeq) were merged to form single “longer-reads” using the SeqPrep pipeline (https://github.com/jstjohn/SeqPrep), with default parameters (a quality score cutoff of phred 33, a minimum merged read length of 15 bp and no mismatches in the overlapping region). Paired-end reads that did not overlap were trimmed using a sliding window approach (window size 10 bases, shift 1 base). Reads were discarded if they were smaller than 30 bases after trimming, and orphan reads were also removed in order to keep pairs only. MIRA v3.4.1 [16] and Trinity [12] assemblers were used independently. Standard assembly metrics, such as number of contigs ≥1 Kb, average contig lengths, and maximum contig size were estimated (Additional file 1: Table S2). Resulting contigs (with a minimum length of 100 bp) derived from both assembly processes were merged into a single file, and those that were redundant were eliminated using the BlastClust program. Redundant contigs were defined as those having greater than 95 % identity over an area covering 95 % of the length of the sequence. Unique contigs were trimmed of low quality, low complexity and poly(A/T) tails using the SeqClean software (http://compbio.dfci.harvard.edu/tgi/), then, they were passed through a second assembly step using the CAP3 assembler [19]. CAP3 was run with default parameters (minimum overlap length of 40 bp and 95 % minimum sequence identity). A set of unigenes from *P. americana* were generated considering only resulting contigs with a minimum size of 200 bp (Additional file 1: Table S2). SOLiD sequence reads were only used for differential expression analysis and they were excluded from the assembly because direct conversion of sequencing reads is possible but not recommended because all bases that follow a single error in colorspace will create errors in all subsequent bases of a read. Additionally, it has been recently reported that a hybrid strategy generated high quality assemblies following three simple recommendations: (1) using a single individual with large representation of biological tissues, (2) merging both long reads and paired-end reads (derived from GS-FLX+ and Illumina platforms, respectively) and (3), using several assemblers in order to combine specific advantage of each [91].

### Annotation of *Persea americana* var. drymifolia unigenes

To annotate sequences obtained by *de novo* assembly, we performed sequence similarity searches using the BLASTX algorithm (*e*-value 10^−3^, bit score ≥25) on *Arabidopsis thaliana* (TAIR v11; http://www.arabidopsis.org/) and *Amborella trichopoda* (http://www.amborella.org/) proteins, and plant proteins from other species available in the Reference Sequence (RefSeq) collection (NCBI; ftp://ftp.ncbi.nlm.nih.gov/refseq/release/plant/). Top protein matches from *Arabidopsis*, *Amborella* or other plant species were assigned to each of the avocado unigenes (Additional file 1: Table S3). The putative protein domains contained within the translated unigenes were identified using Hidden Markov Model (HMM)-based searches against Pfam database (*e*-value 10^−3^) [22]. The gene ontology (GO) functional classes and pathways for each avocado unigene were assigned based on Arabidopsis GO SLIM and pathway annotation (Additional file 1: Table S3). Association of unigenes with KEGG pathways was determined using a directional best hit (BBH) method (that is, top reciprocal BLAST hits) against the Kyoto Encyclopedia of Genes and Genomes database [92]. The KEGG pathways annotation (Additional file 3) was performed in the KEGG Automatic Annotation Server (KAAS) (http:/www.genome.jp/tools/kaas/) [24].

### Expression profile analysis of the *P. americana* transcriptome

After assembling the *P. americana* transcriptome, reads generated by the SOLiD system were separated according to the barcode used, and then were separately aligned to the avocado unigene set using BioScope (Life Technologies). To run whole transcriptome analysis with BioScope, a reference genome is required. Using a custom Perl script, a reference genome proxy was created linking all unigenes derived from the assembly process (in which unigenes were separated by 50 N’s); at the same time, a GTF/GFF file was also created. The libraries were relatively uniform with respect to mapping efficiency (Additional file 4: Table S5). Gene expression levels were calculated by the RPKM (reads per kb per million reads) method, and preferentially expressed genes were selected according to the method described by Stekel [32]. Briefly, all unigenes were submitted to a log-likelihood ratio statistic that trends asymptotically to a *χ*2 distribution, as described by Stekel et al. [32]. We considered as preferentially expressed those unigenes with a value of *R* ≥15 among organs sampled (Additional file 3: Figure S5). This provides a single statistical test to describe the extent to which a gene is differentially expressed between libraries. This method permits identification of differentially expressed genes among any number of libraries. Hierarchical clustering was performed using the Pearson correlation coefficient and average linkage clustering [93]. Results were visualized using GeneSpring GX 7.3.1 software (Agilent Technologies).

### Real-time quantitative PCR verification

Eight avocado unigenes identified as differentially expressed genes were aligned against their Arabidopsis homologues in order to identify the coding sequences in their correct open reading frame. Protein-coding nucleotide sequences were aligned based on their corresponding amino acid translations using the SeaView program (Additional file 4: Table S14). Gene-specific primer pairs (Additional file 4: Table S15), which were designed using the Primer3 v.0.4.0 web tool (http://bioinfo.ut.ee/primer3-0.4.0/primer3/), were used for real-time PCR.

A total of 10 μg of RNA was reverse transcribed for first-strand cDNA synthesis using SuperScript® III Reverse Transcriptase (Life Technologies) according to the manufacturer’s instructions. Reactions were performed with the SYBR Green PCR Master Mix in an ABI 7500 Fast Real-time system. Actin was used as the standard to normalize the content of cDNA, as described previously [94]. The thermal cycling program was set to 95 °C for 5 min, 40 cycles of 95 °C for 30 s, 60 °C for 30 s, and 72 °C for 1 min. Results were analyzed using the ABI 7500 on-board software, version 2.0.5 (Applied Biosystems). The real-time PCR was conducted with at least three experimental replicates for each biological sample.

## Availability of supporting data

All the supporting data are included as additional files, including the sequences of all assembled transcripts. The raw sequencing data was deposited in the NCBI and can be accesed under the following identifier: BioProject PRJNA282441.

## Additional files

Additional file 1: Table S1. Summary of sequencing data generated from *P. americana* transcriptome. **Table S2.** Basic *P. americana* assembly metrics. **Table S3.** Annotation of *P. americana* unigenes. (ZIP 16319 kb) Additional file 2:
*P. americana* unigenes (ZIP 20363 kb) Additional file 3: Figure S1. Complete metabolic network represented in the *P. americana* unigenes. Nodes in this figure are metabolic compounds. Edges are enzymatic transformations. The edges have been highlighted to indicate the modules: energy, carbohydrate and lipid metabolism (green), nucleotide and amino acid metabolism (orange) and genetic information processing (reed). The metabolic network was re-constructed using “Search & Color Pathway” tool from KEGG database (http://www.genome.jp/kegg/). **Figure S2.** Real-time PCR validation of differentially expressed genes. (A) RNA-Seq expression levels measured as reads per kb per million of reads (RPKM). (B) Real-time PCR expression levels given as 40-ΔCT, where ΔCT is the difference in threshold cycle number of the respective gene and the reference ACTIN; the number 40 was chosen because the PCR run stops after 40 cycles and a constant value was required (as calibrator) in order to see the differences existing in three ripening stages (see as example [95]). The results are shown as the averages ± SE of three biological replicates. **Figure S3.** Expression profiles of avocado orthologs to well characterized tomato ripening-associated genes. RNA-Seq expression for 5 different unigenes, measured as RPKM values (y axis) at specific-organs (A) and during fruit ripening (B) are shown. **Figure S4.** Metabolic pathway from palmitic to linoleic acids. The bar graphs show the frequency of transcriptional units as the average of RPKM values of all unigenes annotated as homologs to each of the Arabidopsis genes (represented by red letters in the figure). Avocado organs (to the left) are represented by different colors: fruit (yellow), seed (red), flower (purple), aerial buds (green), leaves (cyan), stem (blue) and roots (pink). Ripening stages (to the right) are shown in green scale (from light to dark; pre-climacteric, climacteric and post-climacteric respectively). This biosynthetic pathway was reconstructed based on information available for *A. thaliana* in BioCyc Database Collection (http://biocyc.org/). **Figure S5.** The number of unigenes for a given value of the test statistic *R* is plotted as a function of *R*. The data falling within 5 ≤ *R* ≤ 15 decrease exponentially with *R*. When *R* ≥15, the number of genes is above this exponential curve. (PPTX 896 kb) Additional file 4: Table S4. Detailed information for unigenes mapping to KEGG pathways in *P. americana*. **Table S5.** Total number of reads mapped by BioScope^TM^ software (LifeTechnologies) against the *P. americana* unigene set. **Table S6** **.** Expression profile matrix of *P. americana* unigenes. **Table S7** **.** Preferentially expressed unigenes in *P. americana* by organ. **Table S8** **.** *P. americana* unigenes selected as organ-specifc. **Table S9** **.** *P. americana* unigenes selected as preferentially expresed in the fruit/flower comparison. **Table S10** **.** Differentially expressed genes during avocado fruit ripening. **Table S11** **.** Association of specific GO terms to preferentially expressed genes involved in the avocado ripening process. **Table S12** **.** Identity percent between avocado (*Persea americana* var. *drymifolia*) and tomato (*Solanum lycopersicum*) genes involved in the ripening process. **Table S13** **.** List of avocado unigenes annotated as homologous to Arabidopsis genes known or suspected to be involved in acyl-lipid metabolism. **Table S14** **.** *P. americana* unigenes annotated as homologous to Arabidopsis genes involved in the fatty acid biosynthetic pathway. **Table S15** **.** Primers employed in real-time PCR assay. (XLSX 15767 kb) Additional file 5: Pairwise sequence alignment (Rippening-like genes) (ZIP 7 kb) Additional file 6: Pairwise sequence alignment (qRT-PCR validation) (ZIP 8 kb)
