# Supplementary material for: Deep sequencing of the Mexican avocado transcriptome, an ancient angiosperm with a high content of fatty acids
Source: BMC Genomics. 2015 Aug 13;16(1):599. doi: 10.1186/s12864-015-1775-y (PMC4533766; doi:10.1186/s12864-015-1775-y)
Supplement: Additional file 3: Figure S1. — Complete metabolic network represented in the P. americana unigenes. Nodes in this figure are metabolic compounds. Edges are enzymatic transformations. The edges have been highlighted to indicate the modules: energy, carbohydrate and lipid metabolism (green), nucleotide and amino acid metabolism (orange) and genetic information processing (reed). The metabolic network was re-constructed using “Search & Color Pathway” tool from KEGG database (http://www.genome.jp/kegg/). Figure S2. Real-time PCR validation of differentially expressed genes. (A) RNA-Seq expression levels measured as reads per kb per million of reads (RPKM). (B) Real-time PCR expression levels given as 40-ΔCT, where ΔCT is the difference in threshold cycle number of the respective gene and the reference ACTIN; the number 40 was chosen because the PCR run stops after 40 cycles and a constant value was required (as calibrator) in order to see the differences existing in three ripening stages (see as example [95]). The results are shown as the averages ± SE of three biological replicates. Figure S3. Expression profiles of avocado orthologs to well characterized tomato ripening-associated genes. RNA-Seq expression for 5 different unigenes, measured as RPKM values (y axis) at specific-organs (A) and during fruit ripening (B) are shown. Figure S4. Metabolic pathway from palmitic to linoleic acids. The bar graphs show the frequency of transcriptional units as the average of RPKM values of all unigenes annotated as homologs to each of the Arabidopsis genes (represented by red letters in the figure). Avocado organs (to the left) are represented by different colors: fruit (yellow), seed (red), flower (purple), aerial buds (green), leaves (cyan), stem (blue) and roots (pink). Ripening stages (to the right) are shown in green scale (from light to dark; pre-climacteric, climacteric and post-climacteric respectively). This biosynthetic pathway was reconstructed based on information available for A. thaliana in BioCy [file 12864_2015_1775_MOESM3_ESM.pptx]

## Slide 1
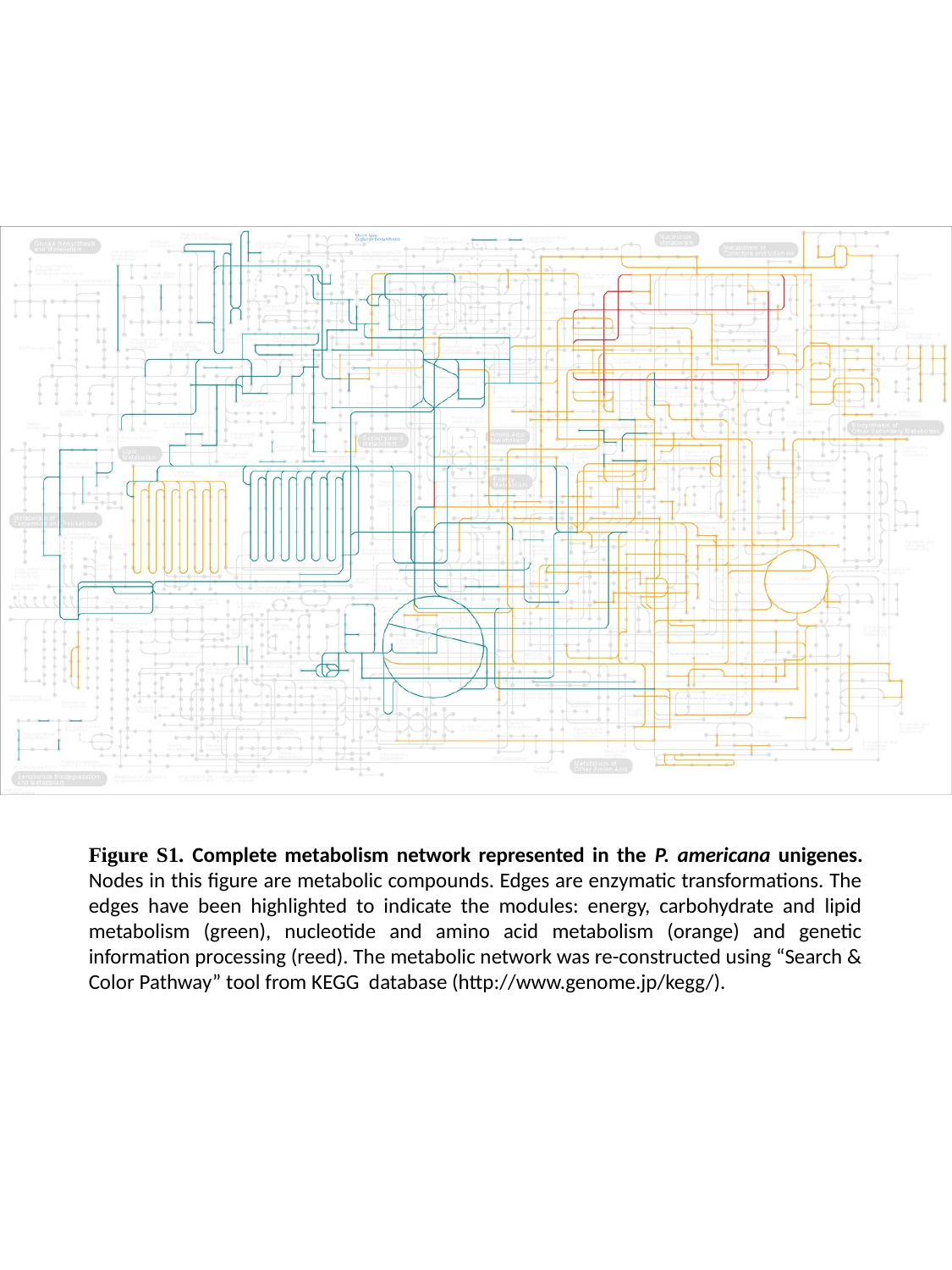

Figure S1. Complete metabolism network represented in the P. americana unigenes. Nodes in this figure are metabolic compounds. Edges are enzymatic transformations. The edges have been highlighted to indicate the modules: energy, carbohydrate and lipid metabolism (green), nucleotide and amino acid metabolism (orange) and genetic information processing (reed). The metabolic network was re-constructed using “Search & Color Pathway” tool from KEGG database (http://www.genome.jp/kegg/).

## Slide 2
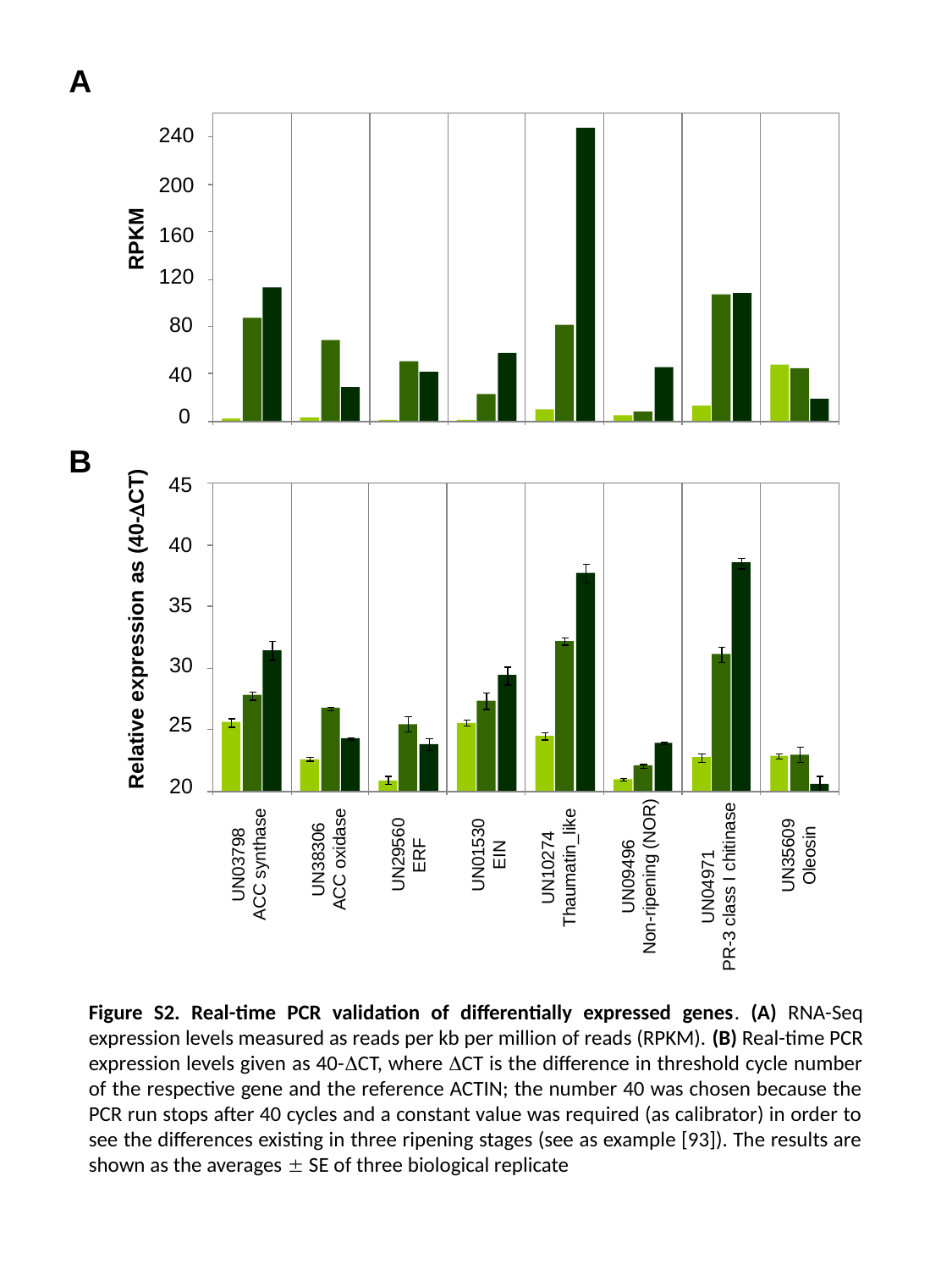

A
240
200
160
RPKM
120
80
40
0
B
45
40
35
Relative expression as (40-ΔCT)
30
25
20
UN29560
ERF
UN01530
EIN
UN35609
Oleosin
UN38306
ACC oxidase
UN03798
ACC synthase
UN10274
Thaumatin_like
UN09496
Non-ripening (NOR)
UN04971
PR-3 class I chitinase
Figure S2. Real-time PCR validation of differentially expressed genes. (A) RNA-Seq expression levels measured as reads per kb per million of reads (RPKM). (B) Real-time PCR expression levels given as 40-ΔCT, where ΔCT is the difference in threshold cycle number of the respective gene and the reference ACTIN; the number 40 was chosen because the PCR run stops after 40 cycles and a constant value was required (as calibrator) in order to see the differences existing in three ripening stages (see as example [93]). The results are shown as the averages ± SE of three biological replicate

## Slide 3
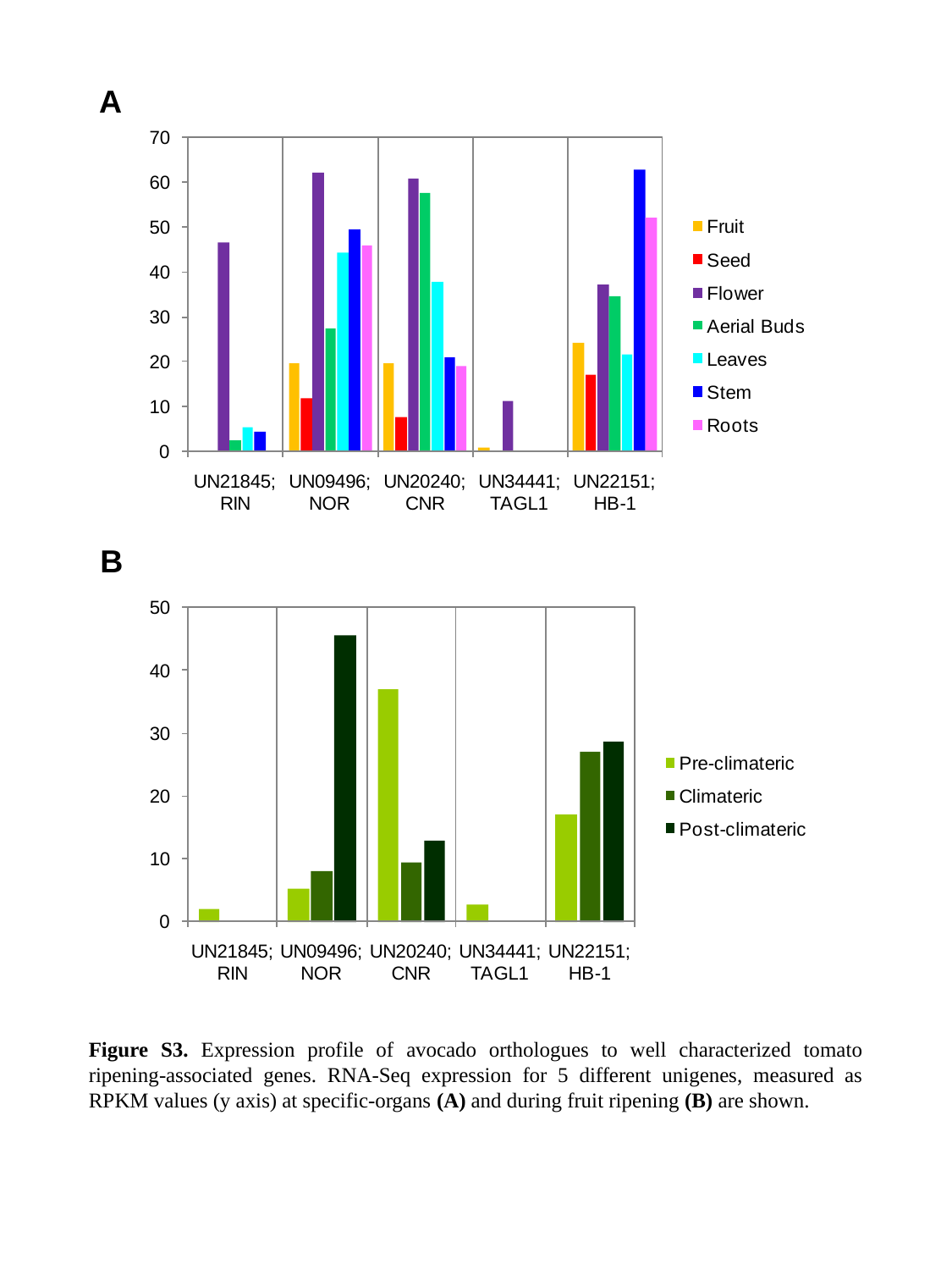

A
B
Figure S3. Expression profile of avocado orthologues to well characterized tomato ripening-associated genes. RNA-Seq expression for 5 different unigenes, measured as RPKM values (y axis) at specific-organs (A) and during fruit ripening (B) are shown.

## Slide 4
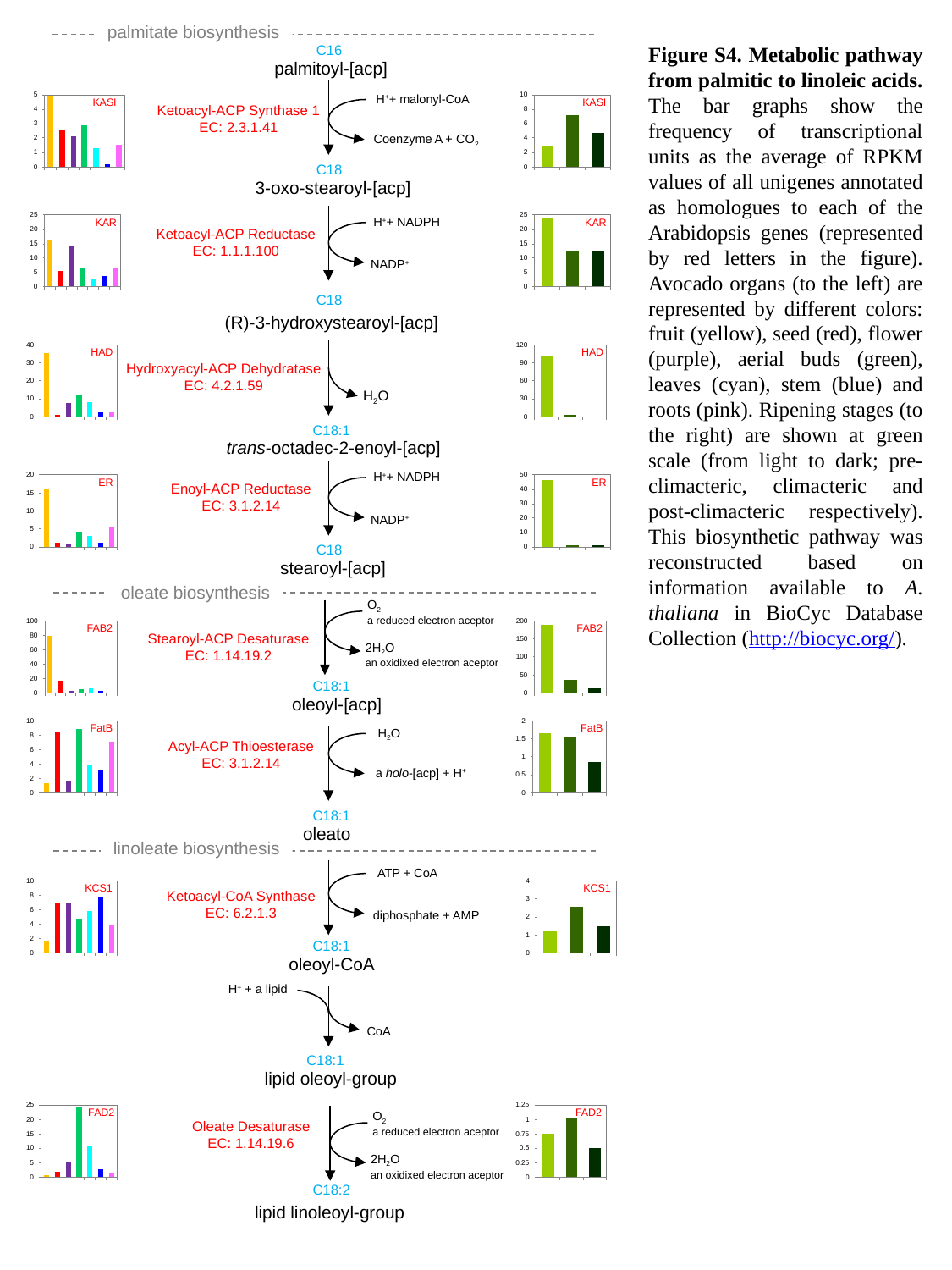

palmitate biosynthesis
Figure S4. Metabolic pathway from palmitic to linoleic acids. The bar graphs show the frequency of transcriptional units as the average of RPKM values of all unigenes annotated as homologues to each of the Arabidopsis genes (represented by red letters in the figure). Avocado organs (to the left) are represented by different colors: fruit (yellow), seed (red), flower (purple), aerial buds (green), leaves (cyan), stem (blue) and roots (pink). Ripening stages (to the right) are shown at green scale (from light to dark; pre-climacteric, climacteric and post-climacteric respectively). This biosynthetic pathway was reconstructed based on information available to A. thaliana in BioCyc Database Collection (http://biocyc.org/).
C16
palmitoyl-[acp]
H++ malonyl-CoA
KASI
KASI
Ketoacyl-ACP Synthase 1
EC: 2.3.1.41
Coenzyme A + CO2
C18
3-oxo-stearoyl-[acp]
H++ NADPH
KAR
KAR
Ketoacyl-ACP Reductase
EC: 1.1.1.100
NADP+
C18
(R)-3-hydroxystearoyl-[acp]
HAD
HAD
Hydroxyacyl-ACP Dehydratase
EC: 4.2.1.59
H2O
C18:1
trans-octadec-2-enoyl-[acp]
H++ NADPH
ER
ER
Enoyl-ACP Reductase
EC: 3.1.2.14
NADP+
C18
stearoyl-[acp]
oleate biosynthesis
O2
a reduced electron aceptor
FAB2
FAB2
Stearoyl-ACP Desaturase
EC: 1.14.19.2
2H2O
an oxidixed electron aceptor
C18:1
oleoyl-[acp]
FatB
FatB
H2O
Acyl-ACP Thioesterase
EC: 3.1.2.14
a holo-[acp] + H+
C18:1
oleato
linoleate biosynthesis
ATP + CoA
KCS1
KCS1
Ketoacyl-CoA Synthase
EC: 6.2.1.3
diphosphate + AMP
C18:1
oleoyl-CoA
H+ + a lipid
CoA
C18:1
lipid oleoyl-group
FAD2
FAD2
O2
a reduced electron aceptor
Oleate Desaturase
EC: 1.14.19.6
2H2O
an oxidixed electron aceptor
C18:2
lipid linoleoyl-group

## Slide 5
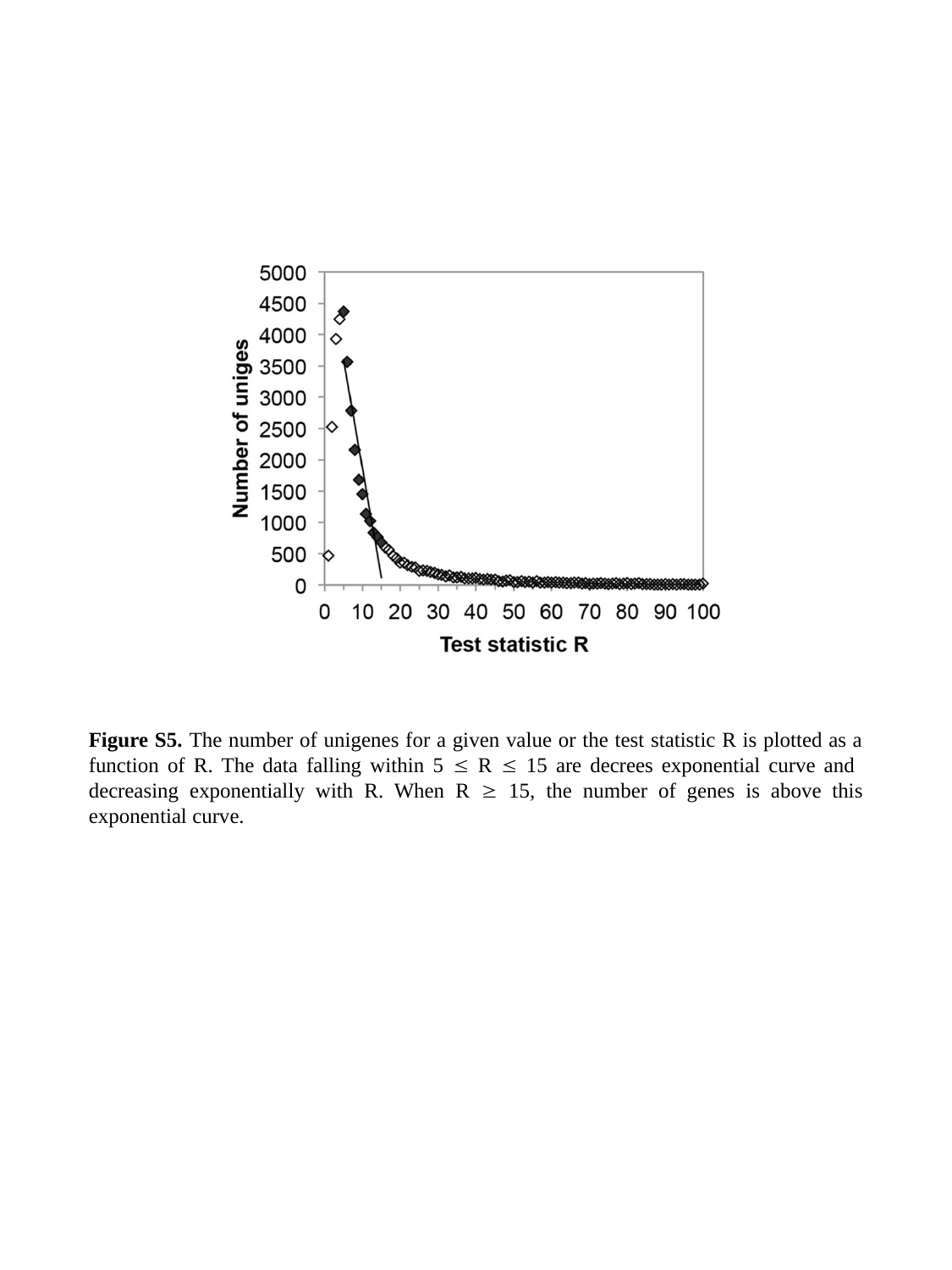

Figure S5. The number of unigenes for a given value or the test statistic R is plotted as a function of R. The data falling within 5  R ≤ 15 are decrees exponential curve and decreasing exponentially with R. When R ≥ 15, the number of genes is above this exponential curve.
